# Occupational burnout syndrome among italian healthcare workers during the COVID-19 pandemic: A real-world study

**DOI:** 10.1192/j.eurpsy.2021.302

**Published:** 2021-08-13

**Authors:** A. Vignapiano, G. Nolfe

**Affiliations:** Struttura Centrale Psicopatologia Da Mobbing E Da Disadattamento Lavorativo, ASL Napoli 1 - Centro, Napoli, Italy

**Keywords:** burn-out, Italian healthcare workers, distress, coviid-19

## Abstract

**Introduction:**

The coronavirus disease 2019 (COVID-19) experience in 2019/2020 carrieda devastating impact on hospital systems and personnel. Therising number of cases, unpreparedness, lack of vital resources, excessiveworkload, and the incapacityto contain the spread has producedincreased psychological and physical pressure among thehealthcare workers. During thepandemic,the extreme pressures experienced by healthcare workers increasedthe risk ofburnout, which has negative consequences for individual health, but also for patient care andthe healthcare system.

**Objectives:**

Our study has been developed with the aims to evaluate the impact of COVID-19 pandemic on occupational burnout of Italian healthcare workers and to identify the presence of protective and the risk factors.

**Methods:**

An online survey addressed the Italian healthcare workers using email invitation, dissemination of the link through social media channels and involvement of professional associations. The snowball sampling procedure gave us the opportunity to recruit a large sample of the Italian healthcare workers with different role, specialties and settings.

**Results:**

During the study period, 5643 responses were recorded. The final sample included 5385 participants. This included 63.2% of medical practitioners, 35.3% of nurses and social workers and 1.5% non-clinical or other. The majority of participants were female (60.7%), 39.4% had at least 20 years of clinical experience and 3170 participants worked in inpatient unit hospital.A subset of participants screened positively for moderate- to-severe symptoms of depression, anxiety. Front-line workers reported high greater severity of psychological distress.

**Conclusions:**

Understanding the health-related consequences of COVID-19 outbreak on Italian healthcare workers is mandatory to provide timely interventions to protect their health.

**Disclosure:**

No significant relationships.

